# Does Terminology Matter When Measuring Stigmatizing Attitudes About Weight? Validation of a Modified Attitudes Toward Obese Persons Scale

**DOI:** 10.21203/rs.3.rs-4208912/v1

**Published:** 2024-04-04

**Authors:** Caitlin A Martin-Wagar, Katelyn A Melcher, Sarah E Attaway, Brooke L Bennett, Connor J Thompson, Oscar Kronenberger, Taylor E Penwell

**Affiliations:** University of Montana; University of Montana; University of Montana; Clemson; University of Montana; University of Montana; University of Montana

**Keywords:** weight stigma, weight bias, anti-fat, stigmatization, obesity

## Abstract

Commonly used medical terms like “obesity” and “overweight” have been identified as stigmatizing. Thus, this study sought to revise a commonly used measure of weight stigmatizing attitudes, the Attitudes Toward Obese Persons (ATOP) scale. We compared the original terminology in the ATOP (e.g., “obese”)to a Modified version using neutral terms (e.g., “higher weight”). We randomized participants (*N* = 599) to either receive the original or Modified ATOP and compared their scores. There was no significant difference between the scores of participants who received the original ATOP and the Modified ATOP, *t*(597) = −2.46, *p* = .550. Through principal component analysis, we found the Modified ATOP is best used as a 13-item unidimensional measure. Findings suggest a Modified version of the ATOP with neutral language is suitable for assessing negative attitudes about higher-weight people without sacrificing psychometric properties. Further examination of the terminology used in weight stigma measures is needed to determine how to best assess weight stigma without reinforcing stigmatizing attitudes. The findings of the present study suggest that the use of neutral terms in measures of anti-fat bias is a promising solution that warrants further investigation.

## Introduction

Weight stigma refers to negative attitudes, beliefs, and stereotypes based on a person’s weight, and is pervasive across education, healthcare, media, and the workplace (Lee et al., 2021; Puhl et al., 2007). Some weight-normative public health campaigns invoke weight stigma and shame in hopes of motivating people to lose weight (Vartarian & Smith, 2013). However, rather than promoting health, weight stigma is associated with numerous adverse mental health outcomes, such as disordered eating, depression, anxiety, stress, body dissatisfaction, poor self-esteem, satisfaction with life, and substance use (Friedman et al., 2005; Haines et al., 2006; Papadopoulos & Brennan, 2015; Puhl & Heuer, 2009; Puhl et al., 2006; Puhl & Lessard, 2020; van Vuuren et al., 2019). Similarly, experienced weight stigma has negative physical health correlates, such as increased cortisol reactivity, avoidance of healthcare, reduced engagement in physical exercise, and arterial pressure (Forhan, Risdon, & Solomon, 2013; Himmelstein et al., 2014; Jackson & Steptoe, 2017; Muennig, 2008; Schvey et al., 2014). Finally, weight stigma is associated with adverse social and academic outcomes, such as rejection by peers and lower classroom engagement (Puhl & Lessard, 2020).

Given the toll of weight stigma, it is important to determine the best ways to measure and address weight stigma. The implications of terminology choice when discussing weight are clear. For people trying to lose weight, the terms “fat,” “obese,” and “extremely obese” used by healthcare providers have been rated as the most undesirable (Puhl et al., 2013). Further, individuals preferred that providers merely referenced individuals’ weight rather than use terms viewed by individuals with higher weight as stigmatizing (Puhl et al., 2013). A systematic review of 33 studies elaborates further; Puhl & Lessard (2020) found a preference for neutral terminology (e.g., “weight”) over terms like “fat” and “obese” across most of these studies. Children have reported feeling upset that their parents described them as “large,” “fat,” “obese,” and “overweight,”; they preferred for their parents to use the terms “healthy weight” or “normal weight” (Puhl et al., 2022). Despite being consistently described by individuals with lived experience as stigmatizing, most current measures of weight stigma still use the medical language (e.g., “obese” and “overweight”) Most of these measures were developed before the stigma of these terms was well understood, suggesting the language they use may be outdated.

Recently, the American Psychological Association recommended using inclusive language when discussing body size and weight (APA, n.d.). They reiterated the points outlined by Meadows & Danielsdottir (2016) that suggest using neutral terms such as “weight” or “higher weight” and suggest psychologists not use person-first language as there is no consensus on whether this is preferred (Liebowitz, 2015). Similarly, the National Institute of Health recommends using “*person with higher weight, higher-weight individual, person with a larger body*, or other similar neutral descriptors, rather than *person with obesity or overweight*” (*NIH, 2023*). Clearly, there is a growing movement to modify the terminology we use to describe people with higher weight. Perhaps, more terminology changes will support a less stigmatizing environment for those with higher weight.

Besides conveying stigma, the term “obese” lacks a solid definition and meaning. In a position statement from the World Obesity Federation, Nutter and colleagues (2023) provide further reason to omit the word in most settings, highlighting the varied obesity definitions proposed by public health organizations. For example, the World Health Organization (WHO; 2024) defines obesity as “abnormal or excessive fat accumulation that presents a risk to health.” Conversely, the CDC defines obesity as a body mass index (BMI) greater than 30.0 (Center for Disease Control and Prevention, 2022). However, a 2022 position statement from the WHO challenges the CDC’s definition, stating that BMI-based approaches to obesity fail to acknowledge the location and function of adipose tissue, as it relates to adiposity-based chronic disease (World Health Organization, 2022). Additionally, Nutter and colleagues (2023) note that definitions of obesity that rely on BMI or other anthropometric metrics fail to capture obesity as a chronic disease. A previous study from Okorodudu and colleagues (2010) supports this position, pointing out that weight and health are not always synonymous and that so-called adiposity-related health impairments can occur in those within the “healthy weight” range (when using BMI as a descriptor of one’s weight-related health).

Given that the term “obesity” is poorly defined and is viewed as stigmatizing, testing Modified versions of these measures is warranted. In our team’s search for measures examining weight stigma toward others that use morefineutral language descriptors (e.g., higher weight, larger-bodied), we could not find any. We noted that the Weight Bias Internalization Scale (Durso & Latner, 2008), a measure of internalized weight bias, had a Modified version that changed the term “overweight” to “my weight,” and maintained good reliability and validity (Pearl & Puhl, 2014). Thus, in the present study, we sought to adapt the Attitudes Towards Obese Persons (ATOP; Allison et al., 1991) scale, a frequently used measure of weight stigma toward others. We compared the original ATOP scale to a Modified version of the instrument, using neutral terms such as “higher weight.” We hypothesized there would be no difference in endorsed negative attitudes toward those with higher weight between those who completed the original ATOP compared to those who completed the Modified version. Finally, given the original ATOP factor structure has been inconsistently replicated (Ambwani et al., 2014; Marek et al., 2021) since its validation study (Allison et al., 1991), we examined the factor structure of both ATOP samples (ATOP original and ATOP Modified). Given the stigmatizing effects of terms like “obese,” this study was conducted in hopes of gathering a better understanding of how weight-related terminology used in a research measure might relate to different attitudes towards individuals of higher weight.

## Method

### Participants and Procedures

Participants were recruited from a university subject pool at a midsized university in the northwestern region of the United States from January 2023 to December 2023. To be eligible to participate, participants needed to be at least 18 years or older. Participants completed a consent form at the beginning of the online Qualtrics survey and were able to receive course or extra credit for their participation. Alternative assignments were provided if students did not want to participate in research. Following recommendations from Tabachnik & Fidell (2019) for sample size for confirmatory factor analysis, we aimed to recruit approximately 300 participants for each randomized group. The university Institutional Review Board approved the study procedures (#185 – 21). Participants were then randomly assigned to complete either the original version of the Attitudes Toward Obese Persons Scale (ATOP; Allison et al., 1991) or a Modified version of the ATOP. After randomization, all participants completed additional questionnaires related to weight bias and eating disorder symptomatology.

### Measures

#### Demographics information.

Participants were asked to report age, gender identity, race, and ethnicity. We also asked participants to provide self-reported anthropometric information (height and weight), and then we calculated body mass index (BMI). Total sample BMI ranged from 16.30 to 72.50 (*M* = 25.07, *SD* = 5.78). There were no significant differences between the ATOP group BMIs (*M* = 25.10, *SD* = 5.81) and ATOP-HW group BMIs (*M* = 25.04, *SD* = 5.76; *t*(596) = 0.12, *p* = .375). Total sample age ranged from 18 to 52 (*M* = 21.23, *SD* = 5.48). There were no significant differences between the ATOP group ages (*M* = 21.38, *SD* = 5.64) and ATOP-HW group age (*M* = 21.09, *SD* = 5.32; *t*(594) = 0.65, *p* = .398). Additional participant demographics for the randomized groups can be found in Table 1.

#### Attitudes Toward Obese Persons Scale (ATOP; Allison et al., 1991).

Negative stereotypical attitudes about people with obesity were measured using the ATOP. The ATOP is a 20-item measure using a Likert rating scale ranging from “I strongly disagree” (−3) to “I strongly agree” (3). A total score is calculated by reverse scoring the 13 negative items, summing the items, and then adding 60. Scores range from 0 to 120, where higher scores indicate more positive attitudes towards people with obesity. There are also three subscales, Different Personality (negative attributes about one’s personality and abilities), Social Difficulties (experiencing social and interpersonal problems), and Self-Esteem. The ATOP has shown adequate internal reliability in adult populations (Allison et al., 1991). In the current study, the ATOP had a McDonald’s Omega of .84, demonstrating good internal consistency.

#### Attitudes Toward Persons with Higher Weight; ATOP-HW).

In the Modified version of the ATOP, the term “obese” was replaced with “higher weight” or “larger body” and “nonobese” was replaced with “thin.” The ATOP-HW had a McDonald’s Omega of .82, demonstrating good internal consistency. See Table 2 for a comparison of wording between the two versions of the ATOP.

### Analytic Plan

Analyses were conducted in IBM^®^ SPSS^®^ Statistics 29 and R. First, descriptive statistics of participant demographics and internal consistency of the measures were examined. Then, we compared the two randomized groups on their total ATOP scores using an independent-sample t-test. Next, two confirmatory factor analyses (CFA) were conducted in R (R Core Team, 2023) with package lavaan (Rosseel, 2012). Model fit was evaluated by the following model fit indices: the comparable fit index (CFI; at least 0.95), the Tucker-Lewis Index (TLI; a least 0.95), the standardized root-mean square error of approximation (RMSEA; 0.06 or below), and the standardized root-mean square residual (SRMR; 0.08 or below; Hu & Bentler, 1999; Thompson, 2004). Finally, an EFA was conducted for the Modified ATOP to determine a suitable factor structure.

## Results

There were 641 students who consented to participate in the study. Of these, two were excluded due to being under the age of 18, six were removed due to stopping the survey immediately after the consent, and 17 were removed due to completing no measures or only a couple of the measures. Attention checks were included throughout the survey (e.g., “select always”). Of the remaining participants, 16 failed at least two of the three attention checks and, as such, were removed. A total of 599 participants remained for analyses.

An assessment of missing data was completed. For the original ATOP, three participants missed one to three items. Their missing items were replaced with the case mean (Parent, 2013). Data was assessed for normality, and no data required transformation or removal.

To assess whether the use of non-stigmatizing language affected ATOP scores, aggregate scores were calculated for participants who received the original ATOP (*N* = 299, *M* = 69.82, *SD* = 16.24) and those who received the Modified version (*N* = 300, *M* = 73.03, *SD* = 15.81). Consistent with the primary hypothesis, there was no significant difference between the scores of participants who received the original version and those who received the Modified version, *t*(597) = −2.46, *p* = .550, *d* = 0.2, 95% CI [−5.69, −0.64].

Next, we performed two confirmatory factor analyses (CFAs) to determine if the factor structure of the ATOP-HW and ATOP samples were consistent with the original ATOP factor structure (Allison et al., 1991). In the original study, Allison and colleagues (1991) identified a three-factor structure (Different Personality, Social Difficulties, and Self-Esteem). In both CFAs we conducted, items were specified to load only on the items’ first-order latent factor. We found a poor model fit for the original ATOP, CFI = 0.747, TLI = 0.712, SRMR = 0.080, RMSEA = 0.084, 90% CI [0.076, 0.093], χ2 (167, *N* = 296) = 517.83, p < .001. We also found a poor model fit for the ATOP-HW, CFI = 0.764, TLI = 0.731, *SRMR* = 0.075, *RMSEA* = 0.079, 90% CI [0.071, 0.087], χ2 (167, *N* = 300) = 480.45, p < .001.

Given the poor model fit for the three-factor solution and the commonly used unidimensional approach with the original ATOP (e.g., Tsai et al., 2019), we conducted principal component analyses (PCAs) to determine an acceptable factor structure for the ATOP-HW. Data was deemed suitable for PCA, with numerous coefficients in the correlation matrix above .3, Kaiser-Meyer-Olkin of .841 (Kaiser, 1974), and a significant Bartlett’s Test of Sphericity (Bartlett, 1954). Five components were identified with eigenvalues > 1, which explained 25.2%, 9.7%, 7.7%, 6.4%, and 5.7% of the variance in the data. However, a clear break in the screeplot was identified after the first factor, most items cross-loaded with component one, and one included less than three items on the component. As such, we explored a unidimensional solution for the ATOP-HW, which is also in line with how most research currently uses the original ATOP. The unidimensional solution explained 25.2% of the variance in the data. Thirteen of the 20 items had factor loadings of at least .45, indicating at least fair loading (Comrey & Lee, 1992). The 13-item ATOP-HW has a good internal consistency (McDonald’s Omega of .84) and the items maintained and removed make theoretical sense (see Table 3 for factor loadings).

## Discussion

The present study aimed to examine the implications of changing the language used to describe individuals in the ATOP measure from “obesity/obese” to “higher weight.” We sought to revise potentially stigmatizing language within a commonly used measure of weight stigma while retaining reliability and validity. The results showed that there were no significant differences in endorsement of weight-based stigma between the two versions of the ATOP (original and Modified). Internal consistency was also good for the ATOP-HW. These findings provide support for a revised version of ATOP used to measurefinegative attitudes toward those with higher weight, without sacrificing psychometric properties.

The original and Modified versions of the ATOP also performed similarly in an examination of their factor structure. The three-factor structure (Different Personality, Social Difficulties, and Self-Esteem) identified in the original ATOP validation study was not replicated in either sample (original or Modified ATOP). Instead, we found a unidimensional structure that retained 13 of the 20 items on the Modified ATOP. These findings are consistent with existing literature as very few studies have successfully replicated the original ATOP three-factor structure (Allison et al., 1991). Of note, most recent studies already use the ATOP as a unidimensional measure of weight-stigmatizing attitudes (Tsai et al., 2019), which our findings support.

In looking at the items removed from the unidimensional modified ATOP, these removed items mostly required respondents to assume the internal feelings or perspectives of those with higher weight (e.g., “Very few higher-weight people are ashamed of their weight,” “Most higher-weight people feel that they are not as good as other people”). It may be that these internal self-esteem items do not consistently fit the other weight stigma items that focus more on negative perceptions of personality and attributes (e.g., “People with very high weight are usually untidy,” “People in larger bodies are just as sexually attractive as thin people”). Future research should continue refining our measurement of weight stigmatizing attitudes, especially as these attitudes may transform with cultural changes throughout time.

Given the stigma of the term“obesity” identified by people with lived experience (Puhl et al., 2013; Puhl et al., 2022) and the inconsistent definitions and uses of “obese” and “obesity” (Nutter et al., 2023; Purnell, 2023), it may be useful for researchers to refine existing measurements of anti-fat weight bias to a more descriptive, neutral term to describe people’s body size. Of note, our examination of revised language includes just some terminology options, and there may be variability among people with lived experience of higher weight in terms of preferred terms (e.g., fat, higher-weight, etc.). As language and perceptions about weight evolve through time, continued exploration of psychometrically sound yet inclusively worded research measures is indicated. Though health organizations are recently encouraging researchers and health care providers to differentiate between higher-weight and higher-weight with adverse health outcomes (e.g., NIH, 2023; World Health Organization, 2022), in our samples, it appears respondents interpreted “obese” and “higher weight” similarly. Future research should consider using qualitative or mixed-methods research to better understand the definitions of each of these constructs in the general population and how these relate to stigmatizing beliefs about those with higher-weight.

Though this research has important implications, there are several limitations to acknowledge and address in future research. First, our sample is a generally age-restrictive, majority White college student population. Future research should consider validating the ATOP-HW in other populations, including healthcare professionals, individuals in larger bodies, and diverse community samples to assess measurement invariance across populations. Further, this study was also strictly quantitative, which means that we do not know how participants conceptualized the individuals described in the measures. Future research should focus on whether any size or weight-related terms (e.g., obese, higher-weight, fat) are interpreted differently for some respondents. Further examination of the terminology used in research contexts is needed to determine how to best assess weight stigma without reinforcing stigmatizing attitudes.

In conclusion, this study provides support for the use of a Modified 13-item ATOP that uses neutral language to describe body size. As researchers aiming to understand and document the harms associated with weight-related bias and discrimination, it is crucial that we do not perpetuate the stigma we are trying to ameliorate in the process. The findings of the present study suggest that the use of neutral terms in measures of anti-fat bias is a promising solution that warrants further investigation. It is vital that we remain critical of our methods and measures, reaffirming their relevance and validity as cultural norms about language and weight continue to change. The present study represents an important first step in this process.

## Figures and Tables

**Table 1:** Demographic Characteristics

	Total Sample *N* = 599		ATOP Sample *n* = 299		ATOP-HW Sample *n* = 300	
Characteristic	*n*	%	*n*	%	*n*	%
Gender Identity						
Cisgender woman	420	70.1	211	70.6	210	70.0
Cisgender man	145	24.2	70	23.4	77	25.7
Nonbinary	16	2.7	10	3.3	6	2.0
Genderfluid	6	1.0	4	1.3	2	0.7
Transgender man	5	0.8	3	1.0	2	0.7
Transgender woman	3	0.5	3	1.0	0	0.0
Demi-girl	2	0.3	1	0.3	1	0.3
Agender	1	0.2	1	0.3	0	0.0
Prefer not to say	1	0.2	0	0.0	1	0.3
Race/ethnicity						
European American/White	530	88.4	265	88.6	263	87.7
Native American/American Indian/Alaska Native/Indigenous	42	7.0	19	6.4	23	7.7
Asian/Asian American	36	6.0	16	5.4	20	6.7
Hispanic/Latino/a/x	34	5.7	21	7.0	13	4.3
African American/Black	8	1.3	7	2.3	1	0.3
Pacific Islander	7	1.2	4	1.3	3	1.0
Middle Eastern/North African	3	0.5	1	0.3	2	0.7
Unknown	1	0.2	1	0.3	0	0.0
Sexual Orientation						
Straight/Heterosexual	446	74.5	223	74.6	223	74.3
Bisexual/Pansexual	107	17.9	54	18.1	53	17.7
Asexual	24	4.0	12	4.0	12	4.0
Lesbian/Gay	17	2.8	8	2.7	9	3.0
Queer	2	0.3	2	0.7	0	0.0
Questioning	1	0.2	0	0.0	1	0.3
Year in College						
Freshman/first year	335	55.9	168	56.2	167	55.7
Sophomore	119	19.9	61	20.4	58	19.3
Junior	77	12.9	38	12.7	39	13.0
Senior	48	8.0	21	7.0	27	9.0
5th year and beyond	20	3.3	11	3.7	9	3.0

* Total percentage for race/ethnicity is beyond 100% because 10.3% of the sample reported being members of two or more categories.

**Table 2:** Wording for the Original ATOP and Modified Version (ATOP-HW)

Original Version	Modified Version
*1. *Obese* people are as happy as *nonobese* people.	*Higher-weight* people are as happy as *thin* people.
2. Most *obese* people feel that they are not as good as other people.	Most *higher-weight* people feel that they are not as good as other people.
3. Most *obese* people are more self-conscious than other people.	Most *higher-weight* people are more self-conscious than other people.
*4. *Obese* workers cannot be as successful as other workers.	*Higher-weight* workers cannot be as successful as other workers.
*5. Most *nonobese* people would not want to marry anyone who is *obese*.	Most *thin* people would not want to marry anyone who has a *larger body*.
*6. Severely *obese* people are usually untidy.	People with *very high weight* are usually untidy.
7. *Obese* people are usually sociable.	*Higher-weight* people are usually sociable.
8. Most *obese* people are not dissatisfied with themselves.	Most people with *higher-weight* are not dissatisfied with themselves.
*9. *Obese* people are just as self-confident as other people.	People with *higher-weight* are just as self-confident as other people.
*10. Most people feel uncomfortable when they associate with *obese* people.	Most people feel uncomfortable when they associate with people with *higher-weight*.
11. *Obese* people are often less aggressive than *nonobese* people.	*Higher-weight* people are often less aggressive than *thin*.
*12. Most *obese* people have different personalities than *nonobese* people.	Most *larger bodied* people have different personalities than *thin* people.
13. Very few *obese* people are ashamed of their weight.	Very few *higher-weight* people are ashamed of their weight.
14. Most *obese* people resent normal weight people.	Most *higher-weight* people resent *thinner* people.
*15. *Obese* people are more emotional than *nonobese* people.	People with *higher-weight* are more emotional than *thin* people.
*16. *Obese* people should not expect to lead normal lives.	People with *higher-weight* should not expect to lead normal lives.
*17. *Obese* people are just as healthy as *nonobese* people.	People with *higher-weight* are just as healthy as *thin* people.
*18. *Obese* people are just as sexually attractive as *nonobese* people.	People in *larger bodies* are just as sexually attractive as *thin* people.
*19. *Obese* people tend to have family problems.	People in *largerbodiestend* to have family problems.
*20. One of the worst things that could happen to a person would be for him to become *obese*.	One of the worst things that could happen to a person would be for *them* to become *higher weight*.

Note. Modified language is shown in italics for ease of the reader. ATOP = Attitudes Toward Obese Persons Scale.

* Indicates the item was retained in the final version of the Attitudes Towards People with Higher Weight Persons Scale

**Table 3:** ATOP-HW Factor Loadings

Item	Factor Loading
**Retained**	
6. People with very high weight are usually untidy.	.66
4. Higher-weight workers cannot be as successful as other workers.	.64
5. Most thin people would not want to marry anyone who has a larger body.	.64
16. People with higher-weight should not expect to lead normal lives.	.63
18. People in larger bodies are just as sexually attractive as thin people.	.62
15. People with higher-weight are more emotional than thin people.	.61
20. One of the worst things that could happen to a person would be for them to become higher weight.	.60
12. Most larger bodied people have different personalities than thin people.	.60
19. People in larger bodies tend to have family problems.	.59
1. Higher-weight people are as happy as thin people.	.54
9. People with higher-weight are just as self-confident as other people.	.52
17. People with higher-weight are just as healthy as thin people.	.49
10. Most people feel uncomfortable when they associate with people with higher-weight.	.48
**Removed**	
14. Most higher-weight people resent thinner people.	.43
3. Most higher-weight people are more self-conscious than other people.	.32
2. Most higher-weight people feel that they are not as good as other people.	.28
11. Higher-weight people are often less aggressive than thin.	.28
8. Most people with higher-weight are not dissatisfied with themselves.	.27
7. Higher-weight people are usually sociable.	.14
13. Very few higher-weight people are ashamed of their weight.	.04

## Data Availability

The datasets generated and/or analyzed during the current study are available in the Open Science Framework repository, https://osf.io/5hcmt.
